# Cascade-activatable NIR-II fluorescent carbonic anhydrase inhibitors for imaging-guided cuproptosis/chemodynamic combination therapy of colorectal cancer

**DOI:** 10.1039/d5sc04668h

**Published:** 2025-08-13

**Authors:** Pu Xu, Yuxin Huang, Gaoyuan Liu, Xuxuan Gu, Xupeng Sun, Yingna Bi, Wen Zhou, Chen Xie, Quli Fan

**Affiliations:** a State Key Laboratory of Flexible Electronics (LoFE) & Institute of Advanced Materials (IAM), Nanjing University of Posts & Telecommunications 9 Wenyuan Road Nanjing 210023 China iamwzhou@njupt.edu.cn iamcxie@njupt.edu.cn iamqlfan@njupt.edu.cn; b College of Chemistry and Chemical Engineering, Qilu Normal University Jinan 250200 China

## Abstract

Colorectal cancer (CRC) is among the malignancies with the highest morbidity, and accurate diagnosis and therapy are essential for improving patient survival. However, CRC is usually diagnosed at an advanced stage, and complete resection of the tumor lesion during treatment is difficult. Herein, we develop a cascade-activatable NIR-II fluorescent inhibitor (Cu@IR783-CAI) for imaging-guided cuproptosis/chemodynamic combination therapy of CRC. Cu@IR783-CAI is synthesized by sequentially modifying a copper complex and a benzenesulfonamide moiety onto NIR-II fluorescent dye IR783. The NIR-II fluorescence signal is quenched by the copper complex *via* a donor-excited photoinduced electron transfer (d-PeT) process under physiological conditions. In the CRC microenvironment, the overexpressed hydrogen sulfide (H_2_S) can react with the copper complex to generate copper sulfide, which hinders the d-PeT process and recovers the NIR-II fluorescence signal. Furthermore, Cu@IR783-CAI targets carbonic anhydrase IX (CA IX), which is overexpressed on the surface of tumor cells, thereby restricting intramolecular rotation and further enhancing the NIR-II fluorescence signal, thus achieving cascade activation. In addition, the copper complex of Cu@IR783-CAI can simultaneously trigger cuproptosis and chemodynamic therapy within tumor cells, demonstrating satisfactory anticancer efficacy both *in vitro* and *in vivo*. Thus, our study provides a smart NIR-II fluorescent CA inhibitor with cascade-activatable features for CRC theranostics.

## Abstract 2

This work reports a cascade-activatable NIR-II fluorescent inhibitor for colorectal cancer therapy. Its fluorescence signal can be sequentially activated, and it can conduct cuproptosis and chemodynamic therapy.

## Graphical abstract

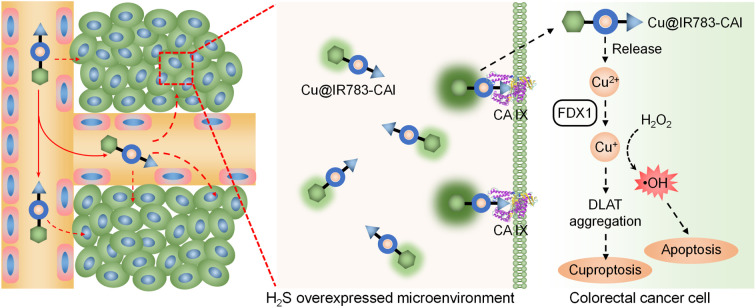

## Introduction

Colorectal cancer (CRC) is among the malignancies with the highest morbidity worldwide.^1–3^ As most patients exhibit few or no symptoms in the early stages and current clinical diagnostic methods have limited ability to detect tiny lesions or metastases, CRC is often diagnosed at an advanced stage.^4^ Currently, surgery is the first choice for CRC treatment, supplemented by other therapeutic approaches including chemotherapy and radiotherapy.^5,6^ However, it is difficult to resect the lesions completely during surgery.^7,8^ In addition, traditional therapeutic modalities such as chemotherapy cannot completely eliminate residual cancer cells, making CRC prone to recurrence and metastasis.^9,10^ Based on the statistical data, more than half of CRC patients at stage IV suffer the issue of recurrence after surgery, significantly affecting the survival rate of patients.^11–13^

Molecular optical imaging techniques have become one of the most promising approaches for disease diagnosis. Among them, the second near-infrared window (NIR-II) fluorescence imaging has been well developed in recent years and shows unique advantages.^14–16^ To date, NIR-II fluorescence imaging has been applied for the diagnosis of tumors, thrombi, atherosclerosis and some inflammation-related diseases, such as liver and kidney injury.^17–20^ In addition, NIR-II fluorescence imaging has been successfully used for surgical navigation in the human body, showing great potential in clinical translation.^21,22^ To further promote the effect of NIR-II fluorescence imaging, a variety of high-performance dyes, especially activatable probes, have been designed.^23–25^ However, restricted by their intrinsic features, it is difficult to develop activatable NIR-II fluorescence probes with high activation folds.^26,27^ As CRC in the early stages is difficult to diagnose, developing a high-performance activatable NIR-II fluorescence probe for CRC imaging is of great significance.^28,29^

Cuproptosis is a novel programmed cell death pathway discovered in recent years, which is highly associated with copper overload within cells.^30–32^ The process of cuproptosis involves the aggregation of lipid-acylated proteins and the loss of iron-sulfur cluster proteins, thus leading to cell death.^33,34^ As a non-apoptotic cell death pathway, cuproptosis can effectively avoid apoptosis resistance, providing an alternative therapeutic approach.^35,36^ To date, the cuproptosis pathway has been demonstrated to be effective in various cancer cell lines, such as breast cancer, lung cancer, and colorectal cancer, and numerous materials have been developed for cuproptosis therapy.^37–39^ In addition, the cuprous ion has been proven to have a much higher Fenton-like reaction efficiency than the ferrous ion.^40,41^ Thus, introducing copper ions not only induces cuproptosis but also enables highly efficient chemodynamic therapy (CDT), providing a better therapeutic efficacy than single therapeutic modalities.^42–45^

**Scheme 1:**
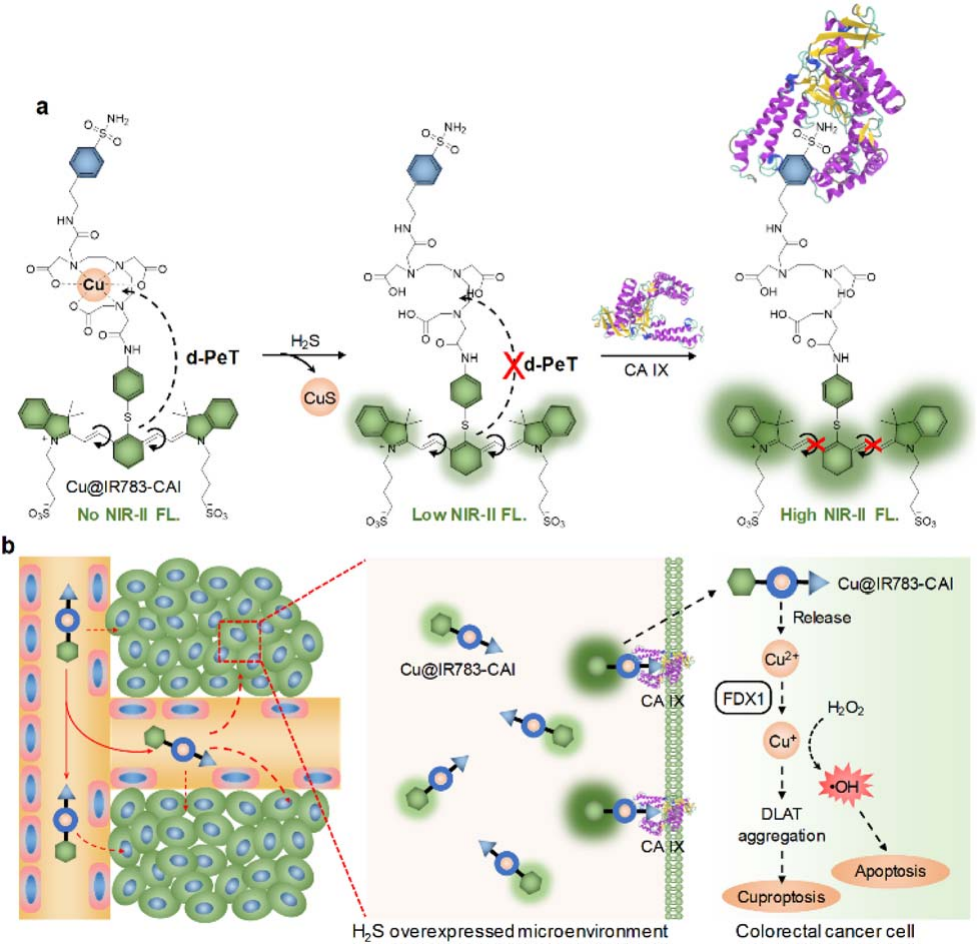
(a) Schematic illustration of the cascade activation process of Cu@IR783-CAI. (b) Schematic illustration of *in vivo* NIR-II fluorescence imaging-guided combination therapy of colorectal cancer mediated by Cu@IR783-CAI.

In this study, we developed a cascade-activatable NIR-II fluorescent carbonic anhydrase inhibitor (Cu@IR783-CAI) for imaging-guided cuproptosis/chemodynamic combination therapy of CRC. Carbonic anhydrases (CAs) are metalloenzymes that catalyze the conversion between carbon dioxide and bicarbonate.^46^ Carbonic anhydrase IX (CA IX) is overexpressed on the surface of a variety of cancer cells, and has been extensively used as a target in cancer treatment.^47^ Cu@IR783-CAI is composed of three main components: a NIR-II fluorescence reporter, a copper complex, and a CA inhibitor (CAI). Under physiological environments, Cu@IR783-CAI showed almost no NIR-II fluorescence signal because of the donor-excited photoinduced electron transfer (d-PeT) process induced by the copper complex. Upon the treatment of hydrogen sulfide (H_2_S), copper ions could form copper sulfide (CuS), and the d-PeT process was eliminated, which led to the enhancement of the NIR-II fluorescence signal. Furthermore, targeting CAs restricted the internal rotation of the double bond in Cu@IR783-CAI, thereby further enhancing the NIR-II fluorescence signal (Scheme 1a). After accumulation in the tumor site, Cu@IR783-CAI could effectively target cancer cells *via* its CA inhibitor group, and its NIR-II fluorescence signal may be greatly enhanced *via* the cascade activation process described above. After being internalized into cancer cells, the released copper ions (Cu^2+^) from Cu@IR783-CAI may be reduced to cuprous ions (Cu^+^) by ferredoxin 1 (FDX1). Cu^+^ could further induce the aggregation of the DLAT gene and cause cuproptosis. Moreover, Cu^+^ was able to react with hydrogen peroxide (H_2_O_2_) to generate highly toxic hydroxyl radicals (˙OH) for CDT (Scheme 1b). Thus, Cu@IR783-CAI is a smart theranostic probe that can specifically target CRC for activatable NIR-II fluorescence imaging and combination therapy.

## Results and discussion

### Synthesis and characterization of Cu@IR783-CAI

Cu@IR783-CAI was synthesized by using a commercially available cyanine dye IR783 as the starting material (Scheme S1). IR783 was first reacted with 4-aminobenzenethiol *via* a substitution reaction to introduce an amino group into the structure of IR783. The obtained IR783-NH_2_ was then reacted with one of the anhydride groups of diethylenetriaminepentaacetic acid (DTPA) dianhydride to conjugate DTPA onto IR783-NH_2_. After that, 4-(2-aminoethyl)benzenesulfonamide was added to react with another anhydride group of the DTPA dianhydride that had been conjugated onto IR783-NH_2_. The obtained compound IR783-CAI was then chelated with Cu^2+^*via* its DTPA moiety to give the final product Cu@IR783-CAI. To study the effect of the benzenesulfonamide group, Cu@IR783 without the benzenesulfonamide group was synthesized as a control (Scheme S2). The chemical structures of Cu@IR783-CAI, Cu@IR783, and all the intermediates were confirmed by proton nuclear magnetic resonance (^1^H NMR) spectra and MALDI-TOF mass spectrometry (Fig. S1–S8). Both Cu@IR783-CAI and Cu@IR783 had good water solubility and exhibited a light green color in aqueous solution.

**Fig. 1:**
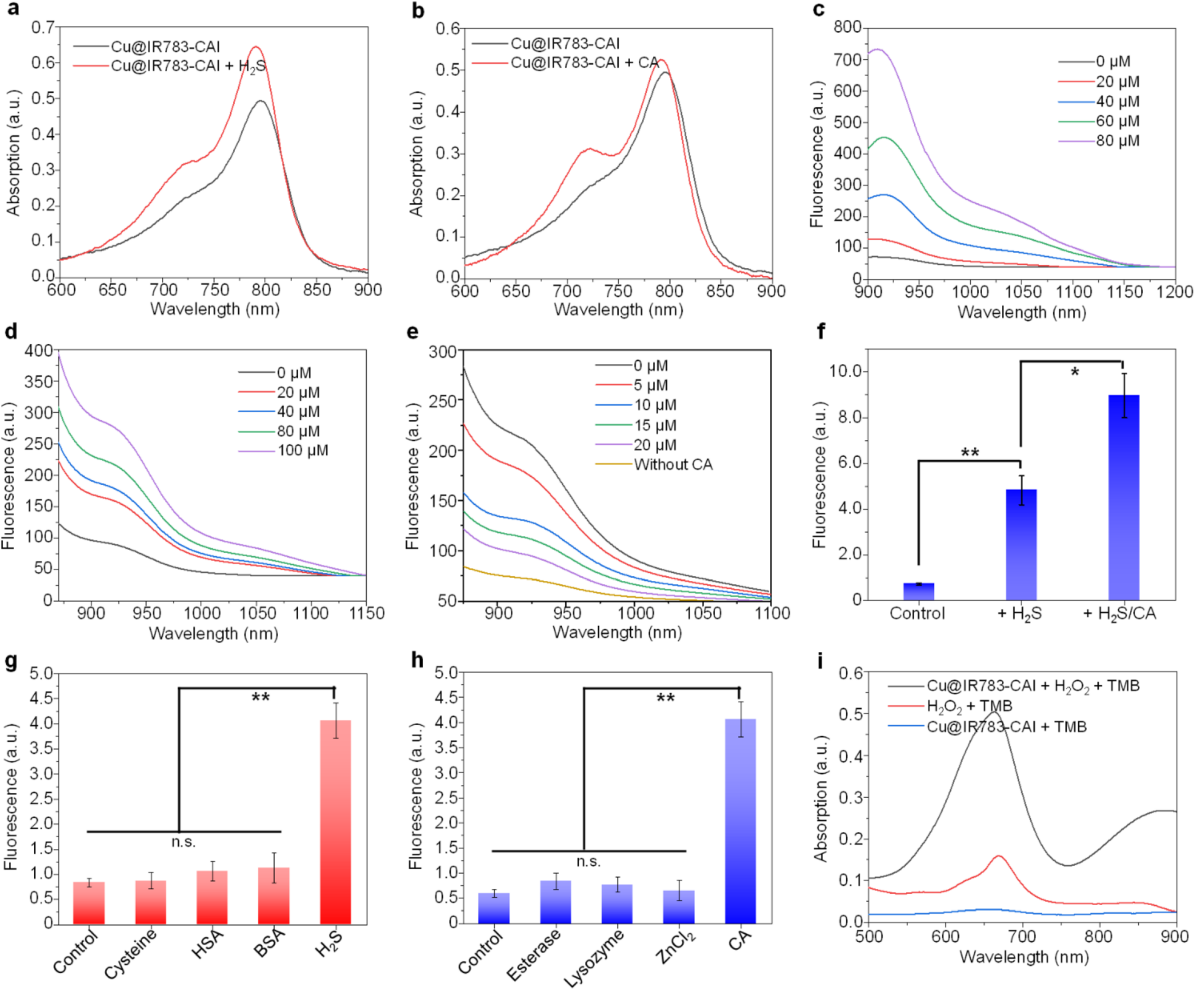
Characterization of Cu@IR783-CAI. Absorption spectra of Cu@IR783-CAI in the presence or absence of H_2_S (a) or CA (b). Fluorescence spectra of Cu@IR783-CAI under the treatment of different concentrations of H_2_S (c) or CA (d). (e) Fluorescence spectra of Cu@IR783-CAI under the treatment of CA with different concentrations of 4-sulfamylbenzoic acid. (f) NIR-II fluorescence intensity of Cu@IR783-CAI under the treatment of H_2_S, CA or H_2_S + CA. (g) NIR-II fluorescence intensity of Cu@IR783-CAI under the treatment of H_2_S and its competitors. (h) NIR-II fluorescence intensity of Cu@IR783-CAI under the treatment of CA and its competitors. (i) Absorption spectra of TMB under different treatments. The error bars represent standard deviations of three separate measurements. n.s.: not significant, *p < 0.05, **p < 0.01.

The optical properties of Cu@IR783-CAI and Cu@IR783 were then studied. Both Cu@IR783-CAI and Cu@IR783 showed intense absorption in the range of 700–850 nm, which was ascribed to the IR783 moiety (Fig. 1a and S9). Upon treatment with H_2_S or CA, the absorption of Cu@IR783-CAI showed no major change, indicating that these stimuli may not damage the chemical structure of the IR783 moiety (Fig. 1b). Both Cu@IR783-CAI and Cu@IR783 had almost no NIR-II emission beyond 1000 nm. With the addition of H_2_S, the NIR-II fluorescence signal of Cu@IR783-CAI increased gradually, and the intensity at 1000 nm was 5.8-fold higher than that before addition under 80 μM of H_2_S (Fig. 1c), and similar fluorescence enhancement was observed for Cu@IR783 (Fig. S10). Such fluorescence enhancement could be attributed to the reaction of copper ions with H_2_S and the formation of CuS, which significantly weakened the d-PeT process between the copper ion and the IR783 moiety. In addition, a linear relationship between H_2_S concentration and NIR-II fluorescence intensity was observed for Cu@IR783-CAI, indicating its capability for quantitative analysis (Fig. S11a). Besides H_2_S, CA was able to greatly enhance the NIR-II fluorescence signal of Cu@IR783-CAI. Under 100 μM of CA, the NIR-II fluorescence signal increased by 2.4-fold compared with the initial value (Fig. 1d), and a linear relationship between CA concentration and fluorescence intensity was observed (Fig. S11b). To study the mechanism of the fluorescence enhancement by CAs, 4-sulfamylbenzoic acid (SA), which is a CAI, was first incubated with CAs, and then Cu@IR783-CAI was added to the inhibited CAs. With the increase in the SA concentration, the NIR-II fluorescence emission of Cu@IR783-CAI decreased gradually, but it remained consistently higher than that in the absence of CA (Fig. 1e). These results showed that the enhancement of the NIR-II fluorescence signal for Cu@IR783-CAI may be attributed to the targeting of the metal center of the CAs, which resulted in the encapsulation of the Cu@IR783-CAI by CAs and the restriction of intramolecular rotation, thus leading to the enhancement of the fluorescence signal.

As H_2_S is overexpressed in the microenvironment of CRC, Cu@IR783-CAI may react with H_2_S first and then with CAs on the surface of the cancer cells. The cascade activation of Cu@IR783-CAI was studied by sequentially adding H_2_S and CAs into Cu@IR783-CAI, followed by measuring its NIR-II fluorescence intensity. The NIR-II fluorescence signal of Cu@IR783-CAI showed a two-step enhancement manner after cascade activation. The fluorescence intensity could be enhanced 6.6 and 1.8-fold after sequentially adding H_2_S and CA, respectively, and the total enhancement could reach 12.4-fold after cascade activation (Fig. 1f). Such high enhancement of the NIR-II fluorescence signal indicated that Cu@IR783-CAI was a good candidate for CRC imaging. After cascade activation, the quantum yield of Cu@IR783-CAI could reach 4.84%. In addition, the Cu@IR783-CAI after cascade activation showed a better photostability than indocyanine green (ICG), which indicated that Cu@IR783-CAI was suitable for tumor imaging (Fig. S12). Except for H_2_S, other biothiols such as cysteine or proteins, including HSA and BSA, may not enhance the NIR-II fluorescence signal of Cu@IR783-CAI (Fig. 1g). Similar results were found in the selectivity study of CA, where other enzymes (esterase, lysozyme), except for CA or the main coordination ion of CA (Zn^2+^), cannot increase the fluorescence signal, confirming the high selectivity of Cu@IR783-CAI (Fig. 1h). It was reasonable to infer that Cu@IR783-CAI could conduct CDT as it contained a copper ion. TMB was used as the hydroxyl radical (˙OH) indicator. After treatment with Cu@IR783-CAI, GSH, and H_2_O_2_, the absorption of TMB at 660 and 880 nm greatly enhanced, which indicated that ˙OH was largely generated under such conditions. In contrast, a much weaker enhancement was observed when Cu@IR783-CAI was absent (Fig. 1i). This result demonstrated that Cu@IR783-CAI was able to trigger the process of CDT in the presence of GSH and H_2_O_2_.

### *In vitro* cellular studies

**Fig. 2:**
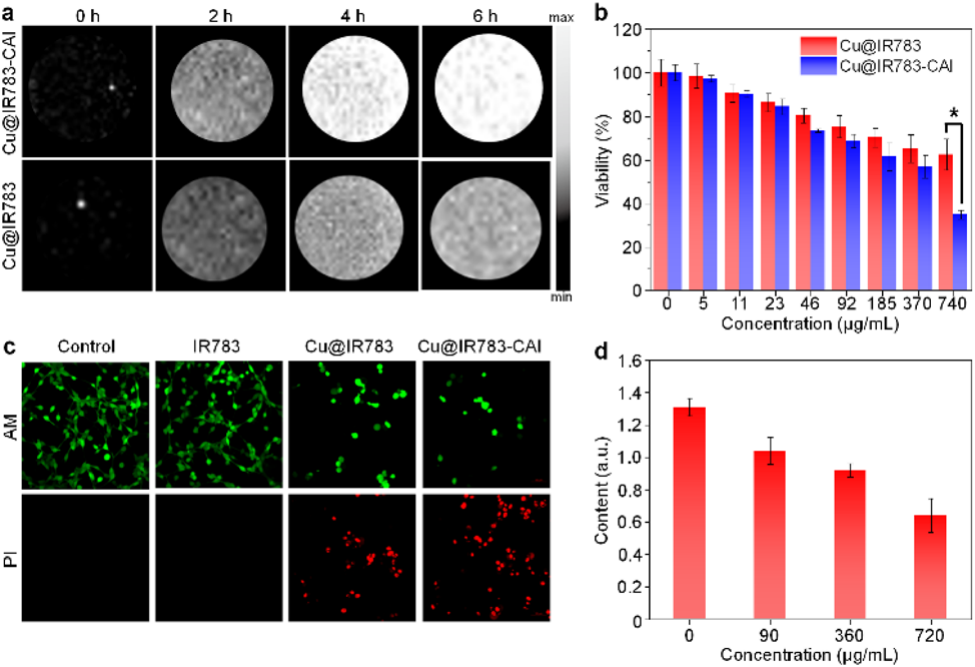
Cell studies. (a) NIR-II fluorescence images of CT26 cells incubated with Cu@IR783-CAI or Cu@IR783 for different times. (b) Viability of CT26 cells under the treatment of different concentrations of Cu@IR783-CAI or Cu@IR783. (c) Live/dead staining of CT26 cells under different treatments. (d) Content of DLAT within CT26 cells under treatment with Cu@IR783-CAI of different concentrations. The error bars represent standard deviations of three separate measurements. *p < 0.05.

The cellular behavior of Cu@IR783-CAI was then studied, and the CRC cell line CT26 was chosen as the model. As the fluorescence emission of Cu@IR783-CAI after activation was too long to be detected by confocal fluorescence imaging, the cellular uptake of Cu@IR783-CAI was studied *via* an *in vivo* NIR-II fluorescence imaging system. It showed that the NIR-II fluorescence signal within Cu@IR783-CAI-incubated cells gradually increased during the incubation time, and the highest fluorescence signal was observed at *t* = 6 h post-incubation (Fig. 2a). At this time point, the fluorescence intensity was 9.2-fold higher than that before incubation (Fig. S13). In contrast, the NIR-II fluorescence signal for Cu@IR783-incubated cells at this time point was only enhanced 6.1-fold (Fig. 2a). This result indicated that Cu@IR783-CAI had a better cellular uptake level than Cu@IR783, and its NIR-II fluorescence signal could be effectively activated within cells. The cytotoxicity of Cu@IR783-CAI was then evaluated by an MTT assay. With increasing concentrations of Cu@IR783-CAI, the viability of CT26 cells decreased gradually. At the concentration of 740 μg mL^−1^, the viability was only 35%, which showed that Cu@IR783-CAI had a satisfactory *in vitro* anticancer effect (Fig. 2b). On the contrary, the viability of cells treated with Cu@IR783 at this concentration was much higher than that of Cu@IR783-CAI-treated cells (62.6% *vs.* 35%). The superior cytotoxicity of Cu@IR783-CAI than Cu@IR783 was further confirmed by live/dead staining. For the cells treated with IR783, almost no dead cells were detected by confocal fluorescence imaging. Approximately half of the cells died after treatment with Cu@IR783, whereas treatment with Cu@IR783-CAI resulted in the death of most cells (Fig. 2c). This result is consistent with the data obtained from the MTT assay. In addition, the flow cytometry analysis showed similar results (Fig. S14). The higher cytotoxicity of Cu@IR783-CAI than Cu@IR783 could be attributed to the better cellular uptake of Cu@IR783-CAI. Cu@IR783-CAI was proven to conduct CDT. Besides CDT, the copper ion was reported to induce cuproptosis, which involved the aggregation of DLAT.^48^ As the concentration of Cu@IR783-CAI increased, the DLAT level within the CT26 cells gradually decreased, reaching 48.8% under treatment with 720 μg mL^−1^ compared with the initial value, indicating that Cu@IR783-CAI may induce the aggregation of DLAT and trigger cuproptosis (Fig. 2d and S15). Overall, these results demonstrated that Cu@IR783-CAI could activate its NIR-II fluorescence signal in CT26 cells and induce CDT and cuproptosis.

### *In vivo* tumor imaging and anti-tumor effects

**Fig. 3:**
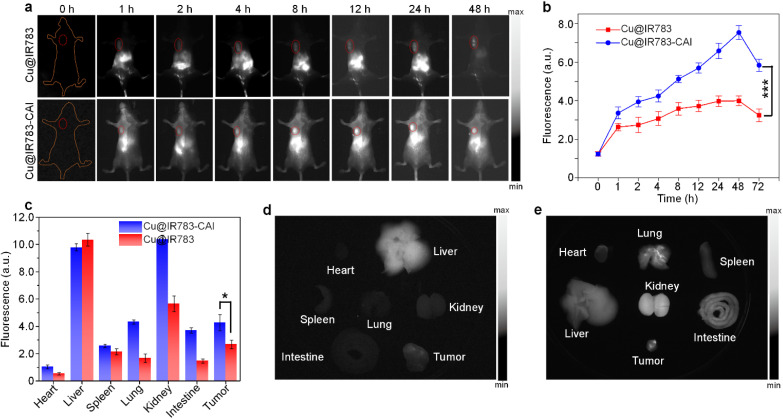
*In vivo* NIR-II fluorescence imaging. (a) NIR-II fluorescence images of tumor-bearing mice i.v. injected with Cu@IR783 or Cu@IR783-CAI at different time points. The red circles indicate the location of tumors. (b) NIR-II fluorescence intensities of the tumor region as a function of post-injection time. (c) NIR-II fluorescence intensities of the major organs collected from mice injected with Cu@IR783 or Cu@IR783-CAI at *t* = 72 h post-injection. NIR-II fluorescence images of the major organs collected from mice injected with Cu@IR783 (d) or Cu@IR783-CAI (e) at *t* = 72 h post-injection. The error bars represent standard deviations of three separate measurements (*n* = 3). *p < 0.05, ***p < 0.001.

As Cu@IR783-CAI showed good responsiveness towards H_2_S and CA, which were both overexpressed in CRC, Cu@IR783-CAI was then applied for *in vivo* imaging of CRC. CT26 cell-inoculated mice were used as a model. The pharmacokinetic properties of Cu@IR783-CAI were first studied in tumor-bearing mice. The results showed that the half-life of Cu@IR783-CAI was 2.9 h, and Cu@IR783-CAI was almost undetectable after 48 h (Fig. S16). Before i.v. injection, the mice showed almost no NIR-II fluorescence signal, which could be attributed to the extremely low tissue background of NIR-II fluorescence imaging. For both Cu@IR783-CAI and Cu@IR783-injected mice, an obvious NIR-II fluorescence signal was detected in the liver region after injection, and the signal became stronger within 24 h (Fig. 3a). This result indicated that both Cu@IR783-CAI and Cu@IR783 were cleared out of the body *via* hepatobiliary metabolism. For Cu@IR783-CAI-injected mice, an obvious NIR-II fluorescence signal was detected in the tumor site within 1 h, while such a signal was clearly detected after 4 h for the Cu@IR783-injected mice. In addition, Cu@IR783-CAI-injected mice showed an obviously higher NIR-II fluorescence signal in the tumor site than Cu@IR783-injected mice at each time point. At *t* = 48 h post-injection, the fluorescence intensity in the tumor of both Cu@IR783-CAI and Cu@IR783-injected mice reached the maximum, and the intensity of the Cu@IR783-CAI-injected mice was 1.9-fold higher than that of the Cu@IR783-injected mice. This result indicated that Cu@IR783-CAI had better tumor targeting capability, probably because of the conjugation of the benzene sulfonamide group (Fig. 3b). After 72 h, the mice were sacrificed, and the major organs were harvested for NIR-II fluorescence imaging. Both Cu@IR783-CAI and Cu@IR783-injected mice showed an NIR-II fluorescence signal in the tumor tissue (Fig. 3c–e). The intensity for the Cu@IR783-CAI-injected mice was 1.6-fold higher than that of the Cu@IR783-injected mice, confirming the better tumor accumulation of Cu@IR783-CAI (Fig. 3c). It was also noticed that the Cu@IR783-CAI-injected mice had a higher NIR-II fluorescence signal in the kidneys and intestines than in Cu@IR783-injected mice, which could be attributed to the expression of CAs in these organs.^49,50^ These results indicated that Cu@IR783-CAI had better tumor targeting and NIR-II fluorescence imaging capability than Cu@IR783.

**Fig. 4:**
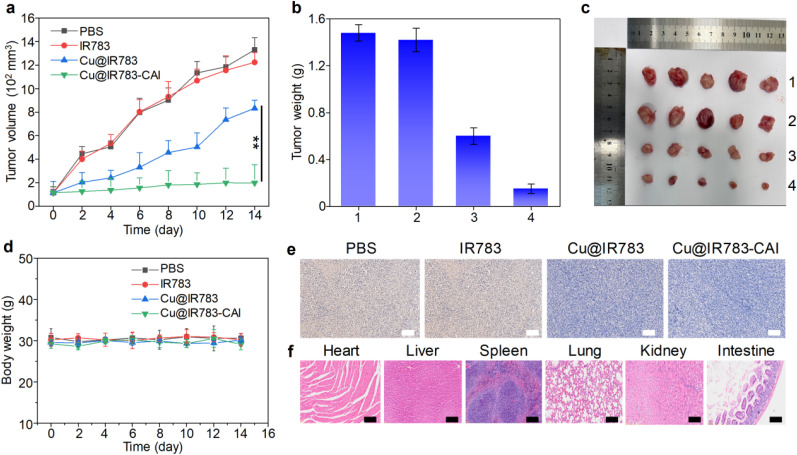
*In vivo* anticancer study. (a) Tumor volume of mice under different treatments as a function of treatment time. Weight (b) and images (c) of tumors collected from different groups after treatment. 1: PBS group; 2: IR783 group; 3: Cu@IR783 group; 4: Cu@IR783-CAI group. (d) Body weight of mice under different treatments as a function of treatment time. (e) PCNA staining of tumor tissue from mice under different treatments. The scale bars represent 50 μm. (f) H & E staining of the major organs collected from mice in the Cu@IR783-CAI group. The scale bars represent 50 μm. The error bars represent standard deviations of five separate measurements (*n* = 5). **p < 0.01.

Owing to its good *in vitro* anticancer efficacy, Cu@IR783-CAI was applied for *in vivo* CRC therapy. CT26 tumor-bearing mice were randomly divided into four groups, which were i.v. injected with PBS, IR783, Cu@IR783, and Cu@IR783-CAI, respectively. The tumor volume of the mice was measured every other day to evaluate the anticancer effect of each group. For the IR783 group, the tumor volume increased rapidly, which was similar to the PBS group, indicating that IR783 had no anticancer effect (Fig. 4a). For the mice injected with Cu@IR783, the tumor growth was inhibited to a certain extent, and the tumor inhibition rate was calculated as 40.9%. Among all the groups, Cu@IR783-CAI had the highest anticancer efficacy, achieving a tumor inhibition rate of 93.1%. After 14 days, the mice were sacrificed, and all the tumors were harvested. The results showed that Cu@IR783-CAI injected mice had the lowest average tumor weight, which was only 10.1% compared with the PBS group (Fig. 4b). In addition, the tumor images indicated that mice from the Cu@IR783-CAI group had the smallest tumor size, further confirming the superior anticancer efficacy of Cu@IR783-CAI (Fig. 4c). During the whole treatment process, the body weight of all the mice remained steady, indicating that all of the treatments had no obvious side effects (Fig. 4d). To further evaluate the therapeutic efficacy and biosafety of the treatments at the cellular level, proliferating cell nuclear antigen (PCNA) and hematoxylin and eosin (H&E) staining of the tumor and organ tissues were conducted, respectively. The PCNA staining showed that most of the tumor cells in the tumor tissue collected from the PBS and IR783-treated mice retained high proliferation activity, while such activity was greatly suppressed in the Cu@IR783 and Cu@IR783-CAI-treated mice, which confirmed the *in vivo* anticancer effect of Cu@IR783-CAI (Fig. 4e). No significant pathological damage was observed in the major organs of the Cu@IR783-CAI-treated mice from H&E staining, demonstrating the biosafety of Cu@IR783-CAI (Fig. 4f). In addition, the good biosafety of Cu@IR783-CAI was also confirmed by a blood test (Fig. S17).

## Conclusions

In summary, we report a cascade-activatable NIR-II fluorescent CA inhibitor (Cu@IR783-CAI) for CRC theranostics. Cu@IR783-CAI was composed of IR783 as the NIR-II fluorescent reporter, a copper complex as the fluorescence quencher, and a benzene sulfonamide moiety as the CA inhibitor. Because of the d-PeT quenching effect of the copper complex, Cu@IR783-CAI showed only a weak NIR-II fluorescence signal. Upon the treatment with H_2_S, H_2_S reacted with the copper complex to form CuS, removing the quenching effect and elevating the NIR-II fluorescence intensity. After activation by H_2_S, Cu@IR783-CAI could further target CAs within the tumor site. This targeting leads to the encapsulation of Cu@IR783-CAI by CAs, which restricts the intramolecular rotation of the IR783 moiety, thus further enhancing the fluorescence intensity. Such cascade activation could elevate the NIR-II fluorescence intensity by 12.4-fold. Because of the modification of the benzenesulfonamide moiety, Cu@IR783-CAI had a higher tumor accumulation than Cu@IR783 without this moiety. In addition, Cu@IR783-CAI could successfully light the tumor up within 1 h. Owing to the presence of a copper complex, Cu@IR783-CAI could induce CDT and cuproptosis both *in vitro* and *in vivo*, achieving a high tumor inhibition rate of 93.1%.

Our work thus provides an effective approach to develop H_2_S/CA cascade-activatable NIR-II fluorescent theranostics. By using other NIR-II fluorescence dyes instead of IR783, probes with a longer emission wavelength could be synthesized. In addition, by conjugating alternative targeting groups, probes for diseases other than tumors can be developed.

## Author contributions

Pu Xu: conceptualization, methodology, writing – original draft. Yuxin Huang: conceptualization, data curation, formal analysis. Gaoyuan Liu: investigation. Xuxuan Gu: investigation. Xupeng Sun: investigation. Yingna Bi: investigation, validation. Wen Zhou: writing – review & editing. Chen Xie: conceptualization, supervision. Quli Fan: supervision, methodology.

## Conflicts of interest

There are no conflicts to declare.

## Supplementary Material

SC-OLF-D5SC04668H-s001

## Data Availability

The data supporting this article have been included as part of the SI. See DOI: https://doi.org/10.1039/d5sc04668h.
