# Supplementary material for: Cascade-activatable NIR-II fluorescent carbonic anhydrase inhibitors for imaging-guided cuproptosis/chemodynamic combination therapy of colorectal cancer
Source: Chem Sci. 2025 Aug 13;16(36):16947–54. doi: 10.1039/d5sc04668h (PMC12379716; doi:10.1039/d5sc04668h)
Supplement: SC-016-D5SC04668H-s001 [file SC-016-D5SC04668H-s001.pdf]

Supplementary Information (SI) for Chemical Science.
This journal is © The Royal Society of Chemistry 2025

Supporting Information

Cascade-activatable NIR-II Fluorescent Carbonic Anhydrase Inhibitors for Imaging-Guided Cuproptosis/Chemodynamic Combination Therapy of Colorectal Cancer

Pu Xu,[a] Yuxin Huang,[a] Gaoyuan Liu,[a] Xuxuan Gu,[a] Xupeng Sun,[a] Yingna Bi,[b] Wen Zhou,[a] Chen Xie,*[a] and Quli Fan*[a]*

[a]State Key Laboratory of Flexible Electronics (LoFE) & Institute of Advanced Materials (IAM), Nanjing University of Posts & Telecommunications, 9 Wenyuan Road, Nanjing 210023, China

[b]College of Chemistry and Chemical Engineering, Qilu Normal University, Jinan 250200, China

Email: iamwzhou@njupt.edu.cn; iamcxie@njupt.edu.cn; iamqlfan@njupt.edu.cn

1. Experimental section

1.1 Chemicals and Materials characterization.

Chemicals. All the chemicals used in the experiments were purchased from Sigma-Aldrich LLC. and used without further purification unless otherwise mentioned.

Characterization. Proton nuclear magnetic resonance (^1H NMR) was conducted on a Bruker Ultra Shield Plus 400 MHz Spectrometer. The mass spectrometry was conducted on an autoflex speed MALDI-TOF MS. Absorption spectra were recorded on a Shimadzu UV-3600 Plus spectrophotometer. Fluorescence spectra were captured on a SENS-9000 (Zolix) fluorescence spectrometer. Confocal fluorescence images were captured on an LSM880 confocal laser scanning microscope (Carl Zeiss, Germany). Flow cytometry analysis was conducted on a Flow Sight Imaging Flow Cytometer (Merck Millipore, Darmstadt, Germany). In vivo NIR-II fluorescence imaging was conducted on a NIR-II fluorescence imaging system (Wuhan Grand-imaging Technology Co., Ltd).

1.2 Synthesis of IR783-NH_2.

IR783 (0.104 g, 0.43 mmol) was dissolved in N,N-dimethylformamide (DMF, 5 mL), followed by the addition of p-aminothiol (0.066 g, 0.528 mmol). The reaction mixture was stirred at 25 °C for 24 h. The resulting solution was precipitated into absolute ether (50 mL) to give the crude product as green solid, and the product was purified by column chromatography (0.121 g, 73% yield). ^1H NMR (400 MHz, Methanol-*d4*) δ 8.88 (d, 1H), 7.46 (d, 2H), 7.42 – 7.38 (m, 2H), 7.35 (d, 2H), 7.25 (t, 2H), 7.04 (d, 2H), 6.66 (d, 2H), 6.34 (d, 2H), 4.66 (s, 1H), 4.19 (t, 4H), 2.10 – 1.88 (m, 12H), 1.41

– 1.34 (m, 4H), 1.33-1.28 (m, 6H). MALDI-TOF-MS: $[M]^+$, calculated m/z = 814.30, found: 814.23.

1.3 Synthesis of IR783-CAI.

Diethylenetriamine pentaacetic (DTPA) anhydride (0.0325 g, 0.0910 mmol) and IR783-NH_2 (0.020 g, 0.0245 mmol) were dissolved in DMF (2 mL). The reaction was carried out at room temperature for 6 h. 4-(2-aminoethyl) benzene sulfonamide (0.018 g, 0.090 mmol) was then added into the solution and the reaction was conducted for another 12 h. The solution was precipitated into anhydrous ether (20 mL) to obtain the crude solid, and then purified by column chromatography to obtain IR783-CAI (0.032 g, 61% of the yield rate). ^{1}H NMR (400 MHz, DMSO-*d6*) δ 8.63 (d, 2H), 7.78-7.65 (m, 2H), 7.60-7.49 (m, 2H), 7.44-7.33 (m, 4H), 7.25 – 7.10 (m, 6H), 6.87 (t, 2H), 6.36 (m, 2H), 5.32 (t, 2H), 5.07 (s, 2H), 4.46 (s, 10H), 4.29 – 4.06 (m, 4H), 3.17 (d, 2H), 2.88 – 2.66 (m, 8H), 2.35-2.17 (m, 4H), 2.05-1.84 (m, 6H), 1.84-1.64 (m, 6H), 1.58-1.38 (m, 10H). MALDI-TOF-MS: $[M]^+$, calculated m/z = 1371.48, found: 1371.02.

1.4 Synthesis of Cu@IR783-CAI.

Cu@IR783 (0.021 g, 0.0153 mmol) was dissolved in DMF (2 mL), followed by addition of copper chloride (0.011 g, 0.0645 mmol) in Tris buffer (pH = 6.8, 0.2 mL). The mixture was stirred at room temperature for 12 h, then dialyzed in pure water for 24 h, and lyophilized to obtain Cu@IR783-CAI (0.0268g with a yield of 86%). ^{1}H NMR (400 MHz, DMSO-*d6*) δ 8.72 (d, 2H), 7.54 (d, 2H), 7.41 (m, 4H), 7.23 (t, 2H), 6.98 (d, 2H), 6.52 (d, 2H), 6.34 (d, 2H), 5.15 (s, 1H), 4.43 (d, 3H), 4.35 (t, 3H), 4.23-4.10 (m, 8H), 3.62 (m, 3H), 3.55 (t, 3H), 3.46 (m, 3H), 3.17 (d, 3H), 2.72 (t, 4H), 1.88

(m, 2H), 1.84-1.67 (m, 8H), 1.53 (s, 12H), 1.24 (s, 4H), 1.16 (s, 1H). MALDI-TOF-MS: $[M]^+$, calculated m/z = 1431.39, found: 1431.25.

1.5 Synthesis of Cu@IR783.

DTPA anhydride (0.0325 g, 0.0910 mmol) and IR783-NH_2 (0.020 g, 0.0245 mmol) were dissolved in DMF (2 mL). The reaction was carried out at room temperature for 6 h. The resulting solution was dialyzed against pure water for 24 h to hydrolyze the remaining anhydride and remove the impurities. The obtained solution was transferred into Tris buffer (pH = 6.8, 2 mL), and copper chloride (0.011 g, 0.0645 mmol) was then added. The reaction was conducted at room temperature for 12 h, then dialyzed against pure water for 24 h, and lyophilized to obtain Cu@IR783. ^1H NMR (400 MHz, DMSO-*d6*) δ 8.61 (d, 2H), 7.94 (s, 1H), 7.53 (d, 2H), 7.40 (m, 2H), 7.22 (d, 2H), 6.35 (d, 2H), 5.73 (s, 2H), 5.31 (t, 2H), 4.49 (t, 2H), 4.23 (q, 18H), 4.16 (t, 2H), 2.89 (s, 2H), 2.80-2.66 (m, 4H), 2.66-2.56 (m, 2H), 2.04-1.84 (m, 4H), 1.83-1.65 (m, 4H), 1.43 (s, 6H), 1.22 (s, 6H), 0.84 (t, 2H). MALDI-TOF-MS: $[M]^+$, calculated m/z = 1249.34, found: 1249.23.

1.6 Responsiveness of Cu@IR783-CAI and Cu@IR783.

The responsiveness of Cu@IR783-CAI and Cu@IR783 towards hydrogen sulfide and carbonic anhydrase (CAs) was investigated. Cu@IR783-CAI or Cu@IR783 solution (0.1 mM) was incubated with different concentrations of hydrogen sulfide (0 mM, 0.2 mM, 0.4 mM, 0.6 mM, 0.8 mM) at 37°C for 30 minutes. Subsequently, the absorption and fluorescence spectra of the solution were tested. To study the responsiveness of Cu@IR783-CAI or Cu@IR783 towards CAs, Cu@IR783-CAI or Cu@IR783 solution

(0.1 mM) was incubated with different concentrations of CAs (0 mM, 0.2 mM, 0.4 mM, 0.8 mM, 1.0 mM) at 37°C for 30 minutes followed by the measurment of optical properties. To study the cascade response of Cu@IR783-CAI and Cu@IR783, hydrogen sulfide and CAs were added in different sequences and their emission spectra were measured. The selectivity study of Cu@IR783-CAI was as follows: Cu@IR783-CAI solution (0.1 mM) was incubated with (Cys, 0.1 mM), bovine serum albumin (BSA, 0.1 mM), human serum albumin (HSA, 0.1 mM), and hydrogen sulfide (0.1 mM) in PBS (0.01 M, pH=7.4) at 37°C for 30 minutes, respectively. After incubation, the absorption and fluorescence spectra of Cu@IR783-CAI were detected. Then, Cu@IR783-CAI solution (0.1 mM) was incubated with bacterial enzymes (0.1 mM), esterases (0.1 mM), zinc chloride (0.1 mM), and carbonic anhydrase (0.1 mM) in PBS at 37°C for 30 minutes, respectively. After incubation, the absorption and fluorescence spectra of Cu@IR783-CAI were detected.

1.7 Cytotoxicity assay.

CT26 cells were purchased from Jiangsu KeyGEN Biotech Corp., Ltd, and the cells were cultured in DMEM medium supplemented with 10% fetal bovine serum (FBS), 100 U/mL penicillin and 100 μg/mL streptomycin at 37°C in a 5% CO_2 humidified atmosphere. The cells were inoculated into 96-well plates with a density of 8500 cells per well and incubated for 24 h. Then the cell culture medium was replaced with 200 μL of fresh DMEM cell culture medium with Cu@IR783-CAI or Cu@IR783 (at concentrations of 0 μg/mL, 2 μg/mL, 4 μg/mL, 8 μg/mL, 16 μg /mL, 32 μg/mL, 64 μg/mL) and incubated for another 20 h. The cell culture medium was then removed

and the cells were washed carefully with PBS. Then 120 μL of fresh DMEM cell culture medium containing 20 μL of 3-(4,5-dimethylthiazol-2)-2,5-diphenyltetrazolium bromide (MTT) was added to each well, and incubated for another 4 h. The absorbance at 490 nm was measured in each well using a Hiscan XM Micro CT zymometer to calculate the cell viability.

1.8 Cellular uptake.

The cellular uptake was studied by using in vivo NIR-II fluorescence imaging system. CT26 cells were inoculated in cell culture dishes with a density of 5×10^4 cells per dish and incubated for 24 h. After incubation with Cu@IR783-CAI or Cu@IR783 for 2 h, 4 h and 6 h, the cells were digested and mixed with PBS. NIR-II fluorescence signals of cells were then collected by in vivo NIR-II fluorescence imaging system under 808 nm laser irradiation with a 980 nm long-pass filter.

1.9 Live/Dead Cell Staining Assay.

CT26 cells (approximately 5×10^4) were seeded in cell culture dishes and incubated for 24 h, then randomly divided into four groups: (1) Control group, (2) IR783 group, (3) Cu@IR783 group, and (4) Cu@IR783-CAI group. For live/dead cell staining, CT26 cells were co-incubated with Cu@IR783-CAI or Cu@IR783/IR783 (740 μg/mL) for 24 h, while CT26 cells without materials addition were served as the control. Subsequently, the medium was removed, and the cells were washed three times with cold PBS buffer. Fresh PBS (1 mL) was added to each dish, followed by the addition of 1 μM Calcein AM and 1 μM propidium iodide (PI), and incubated for 30 minutes.

After removing PBS and washing with fresh PBS for three times, fluorescence images of Calcein AM and PI were acquired by LSM880 confocal laser scanning microscope.

1.10 Apoptosis Assessment by Flow Cytometry.

CT26 cells (approximately 5×10^4) were seeded in 6-well plates and cultured at 37°C for 24 hours, then randomly divided into four groups: (1) Control group, (2) IR783 group, (3) Cu@IR783 group, (4) Cu@IR783-CAI group. After discarding the culture medium, fresh DMEM medium (1.5 mL) containing Cu@IR783-CAI (740 μg/mL), Cu@IR783 (740 μg/mL), or IR783 (740 μg/mL) was added to groups, respectively. Untreated CT26 cells were served as the control group. After further incubation at 37°C for 12 h, the medium was discarded, and the cells were washed with PBS. Subsequently, the medium was removed, and the cells were digested with trypsin, and resuspended in 0.2 mL PBS for Annexin V-FITC/PI double staining. After staining, apoptotic cell populations were analyzed by Flow Sight imaging flow cytometer, with 10,000 live cells examined per group.

1.11. Tumor mouse model.

All the mice experiments were carried out in accordance with the guidelines of the Laboratory Animal Center of Jiangsu KeyGEN Biotech Corp., Ltd and approved by the Animal Ethics Committee of Simcere BioTech Corp., Ltd. The approval number was IACUC-004. 1 million CT26 cells were inoculated at the flank region of the right arm of balb/c mice to establish the tumor model. The volume of tumor was calculated as follows:

$$V = Dd^2/2$$

V represents the volume of tumor, D and d represent maximum and minimum diameter of tumor, respectively.

1.12 In vivo NIR-II fluorescence imaging.

When the tumor volume reached 100 ~ 120 mm^3, Cu@IR783-CAI or Cu@IR783 (4 mg/kg) were intravenously (i.v.) injected into the tumor bearing balb/c mice . NIR-II fluorescence imaging was performed by In vivo NIR-II fluorescence imaging system under 808 nm laser irradiation, and the signals were collected through a 980 nm long-pass filter at designated time points. The mice were then euthanized when the fluorescence signal in the tumor site decreased, and the biodistribution of probes was further recorded via NIR-II fluorescence imaging.

1.13. In Vivo Anticancer Study.

CT26 tumor-bearing BALB/c mice were randomly divided into 4 groups, with each group consisting of 5 mice. The mice in each group were intravenously injected with PBS (100 μL), IR783 (100 μL, 400 μg/mL), Cu@IR783 (100 μL, 400 μg/mL), and Cu@IR783-CAI (100 μL, 400 μg/mL), respectively. Tumor volumes and body weights of the mice were measured every other day. After 14 days, the mice were sacrificed and the tumors were collected and photographed. For histological studies, tumor tissues collected from each group and major organs from Cu@IR783-CAI group were performed PCNA and H&E staining, respectively.

1.14 Data Analysis.

Statistical calculations were performed using GraphPad Prism software. Data are presented as mean ± SD unless otherwise mentioned. For continuous variables,

between-group differences were analyzed using: (1) two-tailed Student's t-test for dual-group comparisons, or (2) one-way ANOVA with Tukey's post hoc tests for multi-group comparisons (≥3 groups). Statistical significance was defined as $P \leq 0.05$.

2. Supporting figures

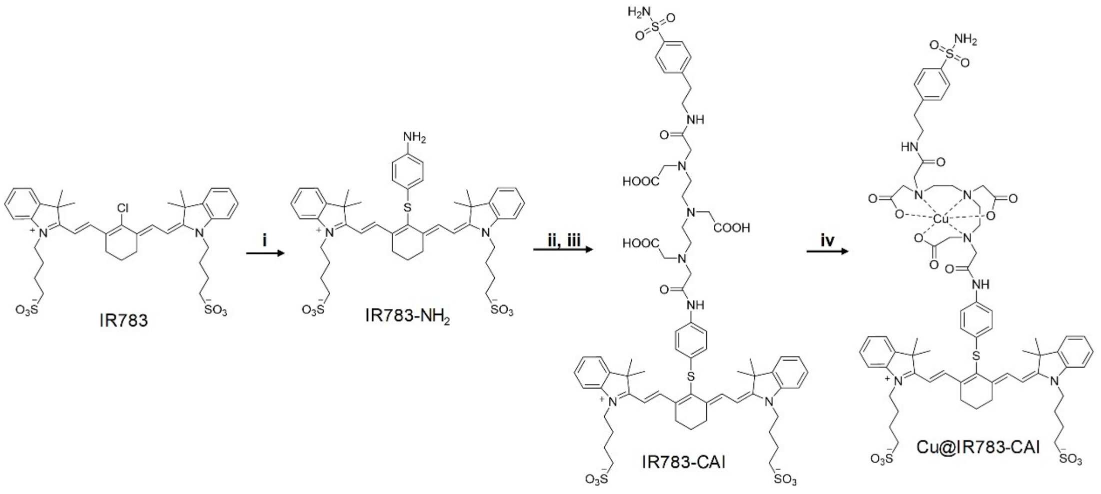

Scheme S1. Synthetic route of Cu@IR783-CAI. Reagents and conditions: i) p-aminothiol, DMF, 25 °C, 24 h; ii) DTPA anhydride, DMF, 25 °C, 12 h; iii) 4-(2-aminoethyl) benzene sulfonamide, DMF, 25 °C, 12 h; iv) copper chloride, DMF/tris buffer, 25 °C, 12 h.

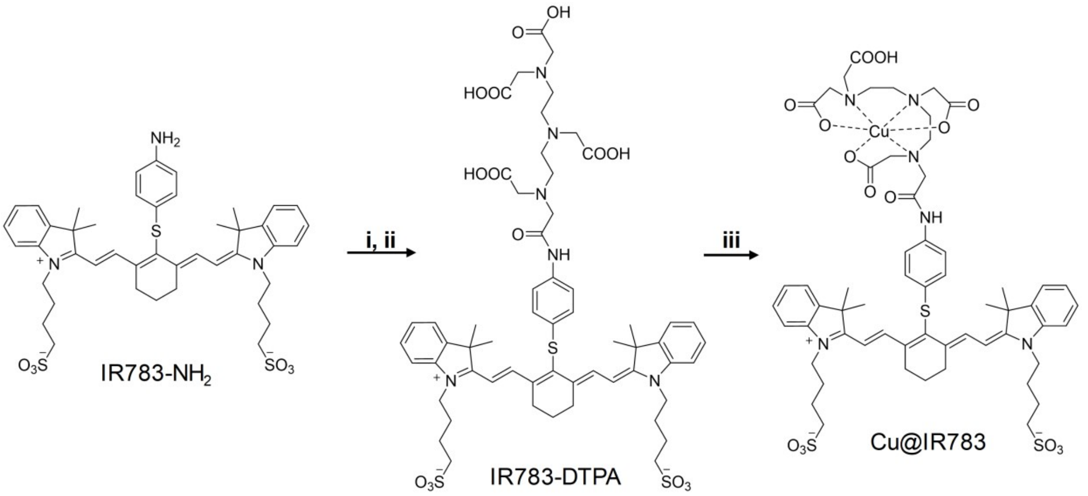

Scheme S2. Synthetic route of Cu@IR783. Reagents and conditions: i) DTPA anhydride, DMF, 25 °C, 12 h; ii) DI water, 25 °C, 12 h; iv) copper chloride, DMF/tris buffer, 25 °C, 12 h.

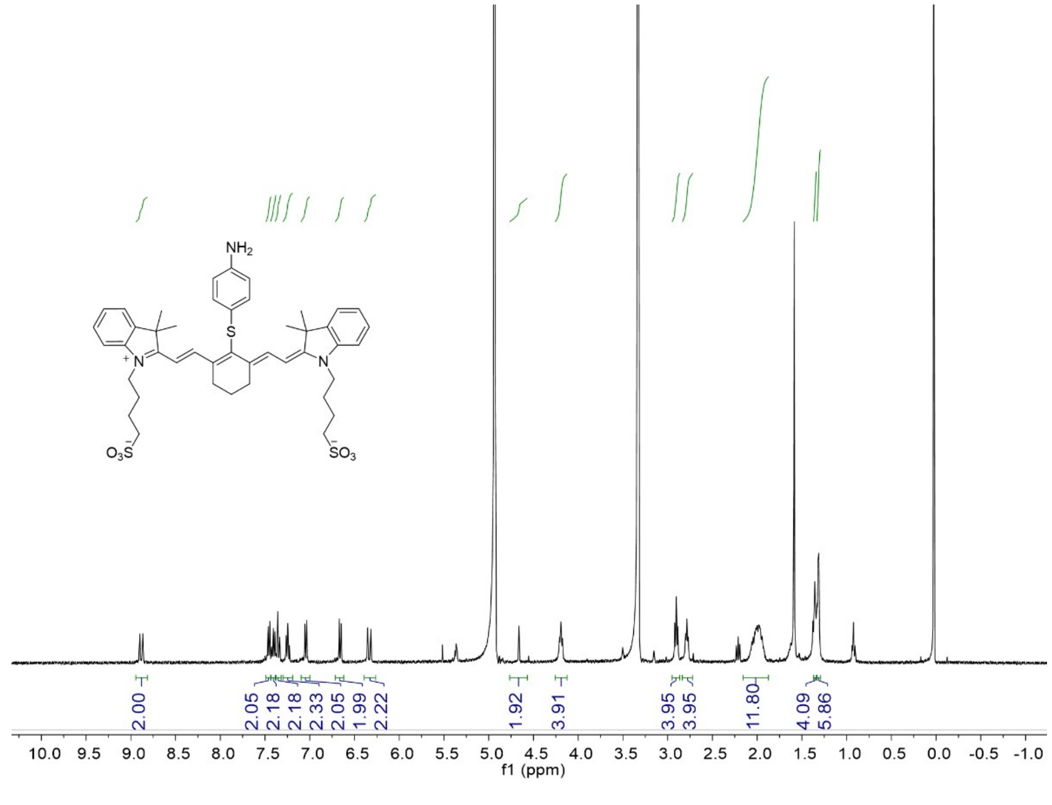

Figure S1. ^1H NMR spectrum of IR783-NH_2. MeOH-*d4* was used as the solvent.

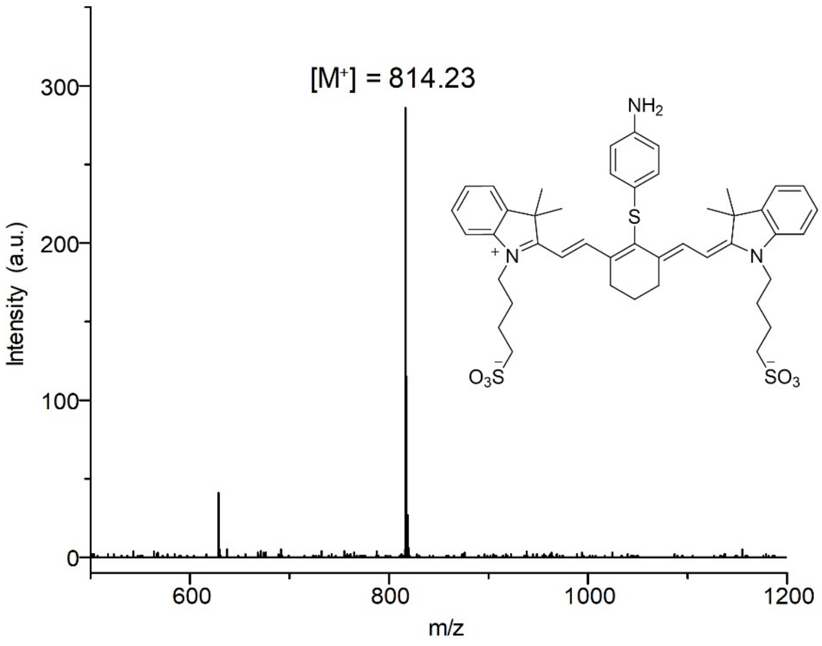

Figure S2. MALDI-TOF-MS of IR783-NH_2.

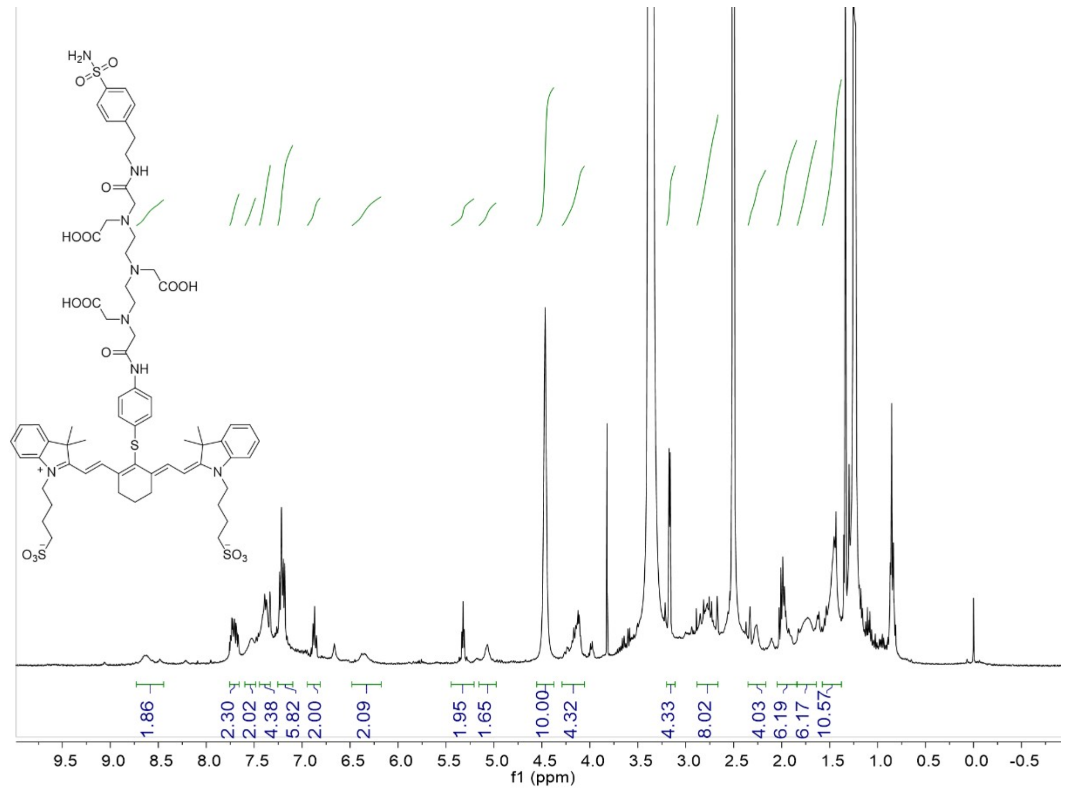

Figure S3. ^{1}H NMR spectrum of IR783-CAI. DMSO-*d6* was used as the solvent.

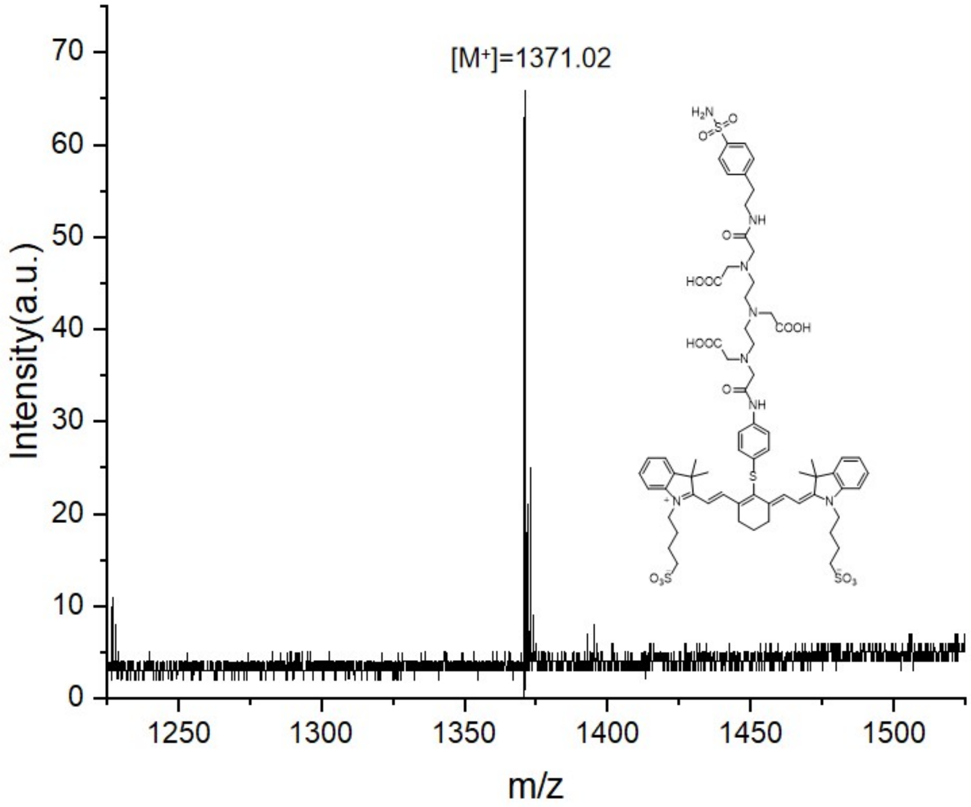

Figure S4. MALDI-TOF MS of IR783-CAI.

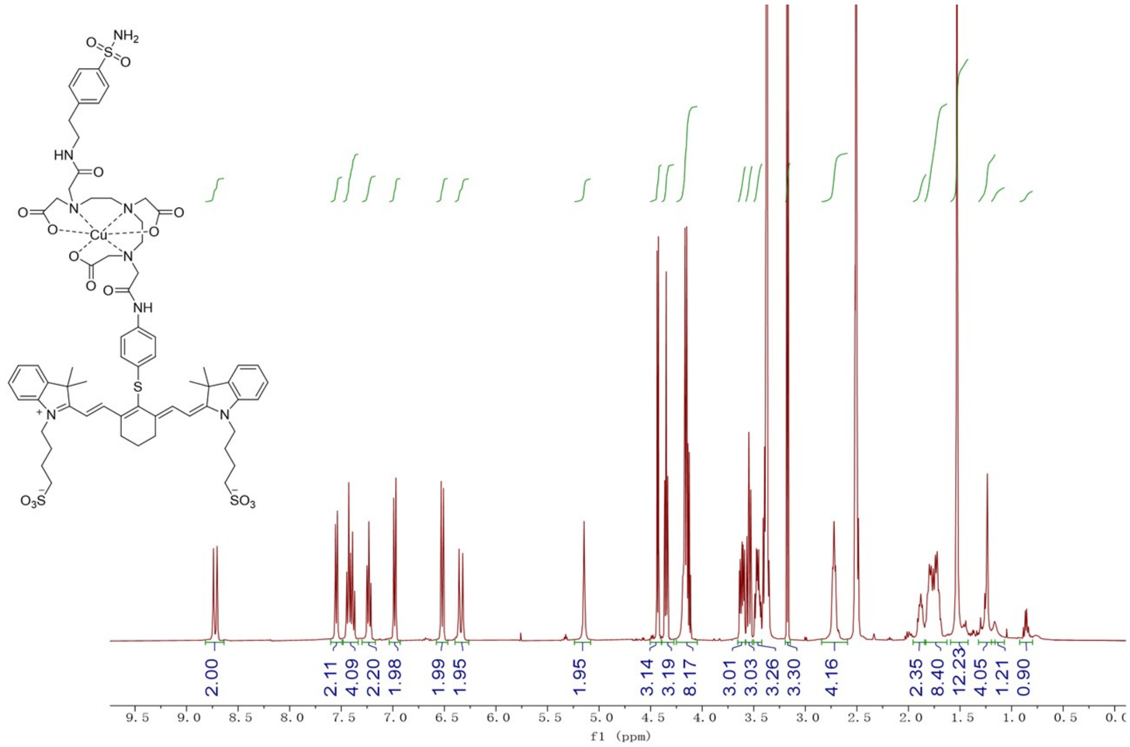

Figure S5. ^{1}H NMR spectrum of Cu@IR783-CAI. DMSO-*d6* was used as the solvent.

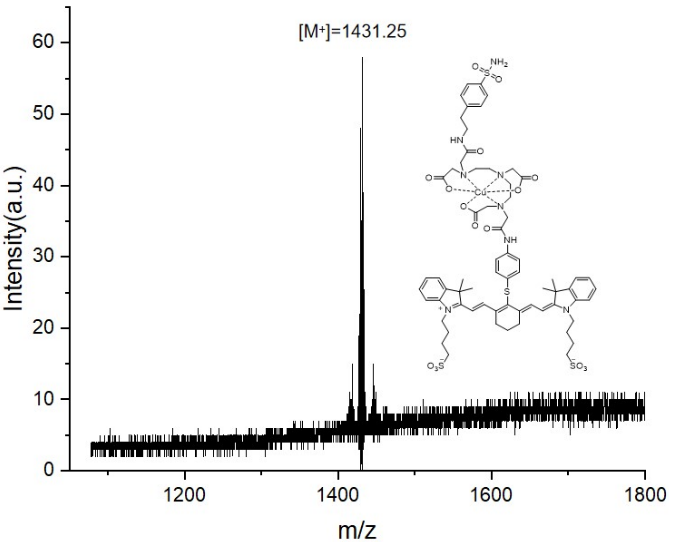

Figure S6. MALDI-TOF MS of Cu@IR783-CAI.

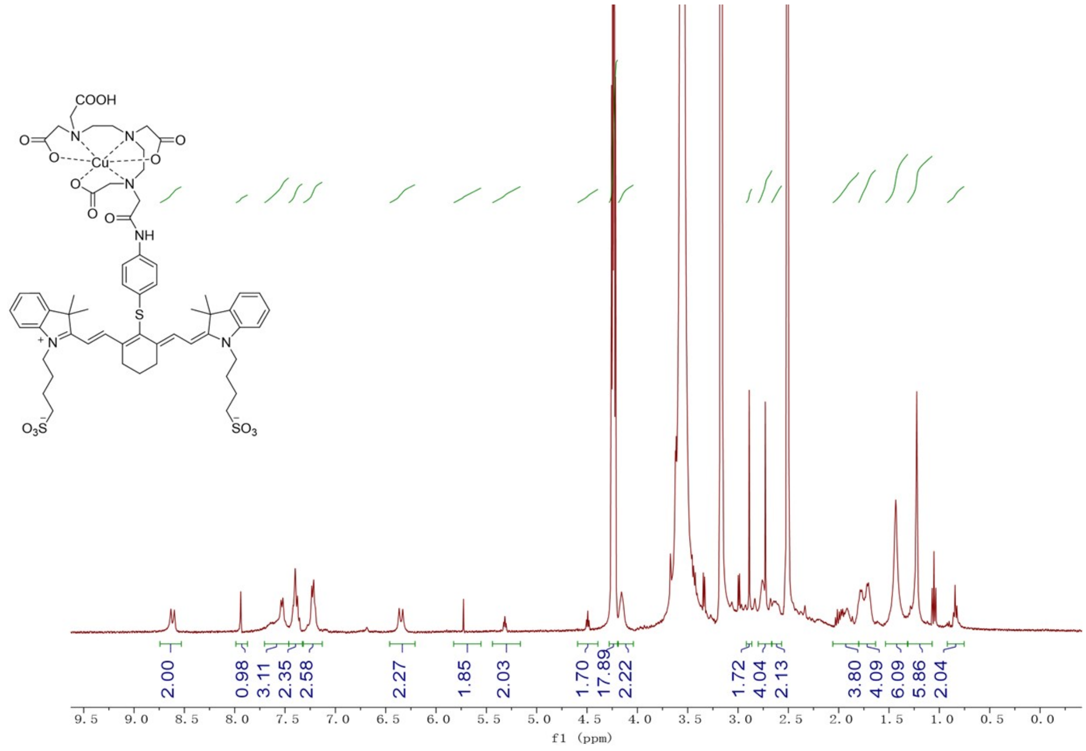

Figure S7. ^{1}H NMR spectrum of Cu@IR783. DMSO-*d6* was used as the solvent.

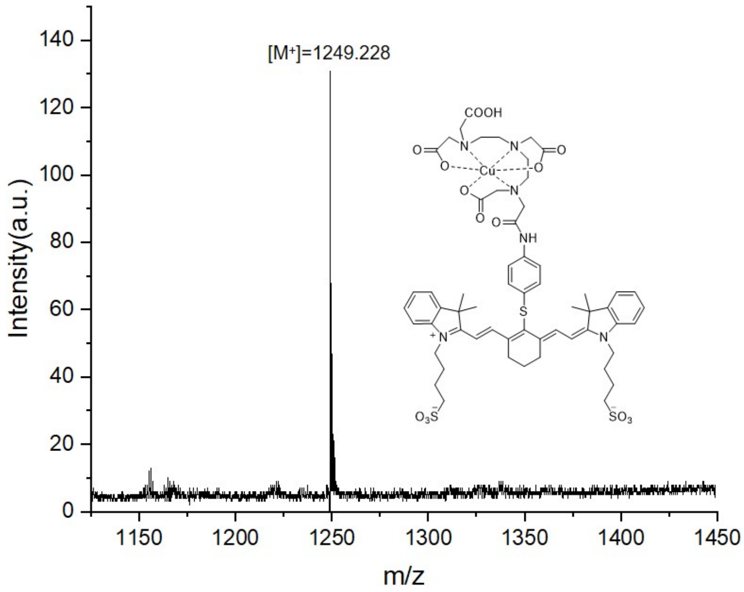

Figure S8. MALDI-TOF MS of Cu@IR783.

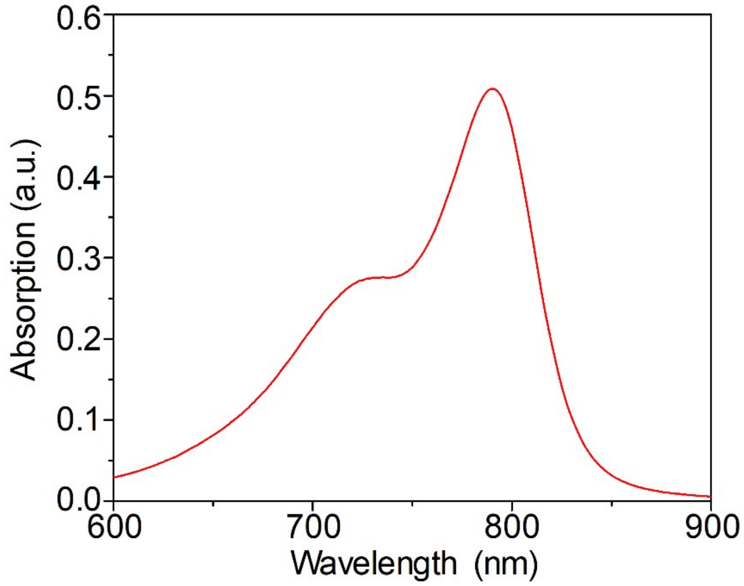

Figure S9. Absorption spectrum of Cu@IR783.

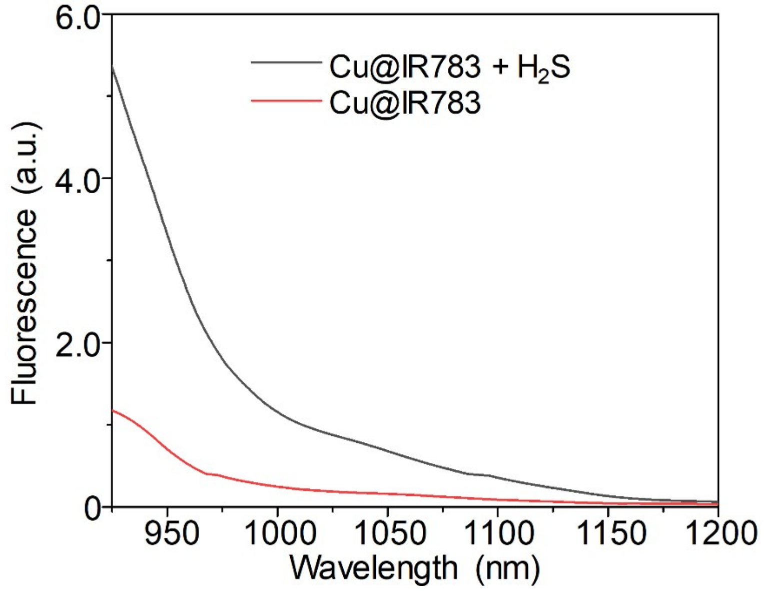

Figure S10. Fluorescence spectra of Cu@IR783 with or without addition of H_2S.

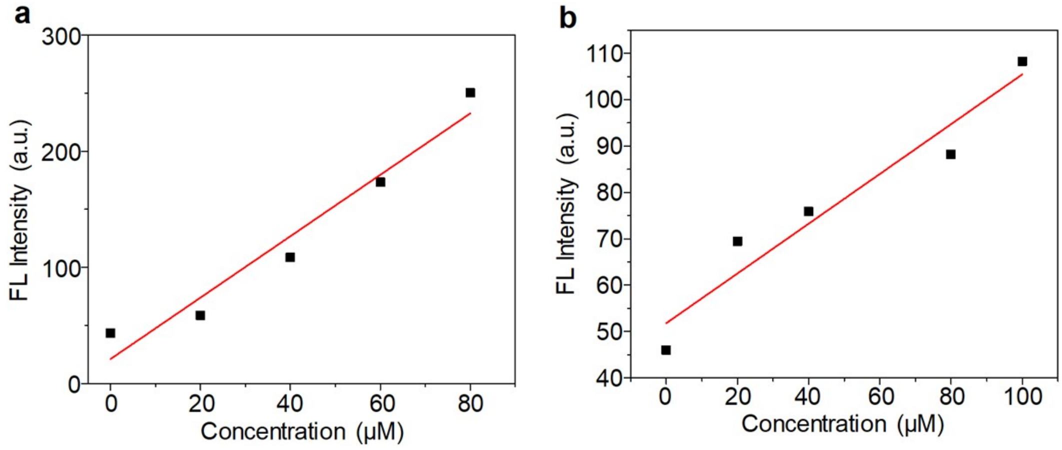

Figure S11. NIR-II fluorescence intensity of Cu@IR783-CAI as a function of H_2S (a) and CA (b) concentration.

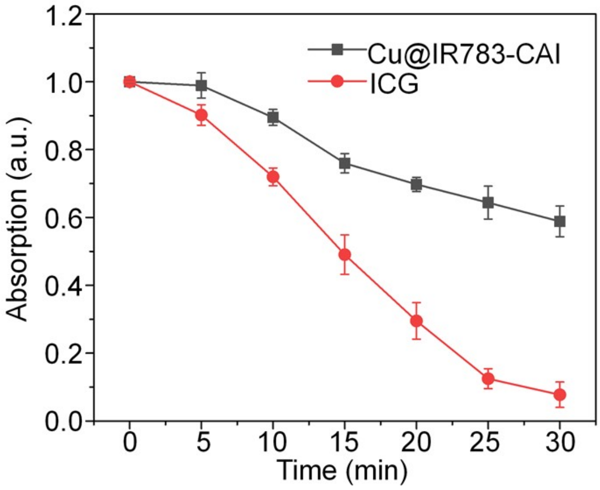

Figure S12. Normalized absorption changes of Cu@IR783-CAI and ICG as a function of irradiation time.

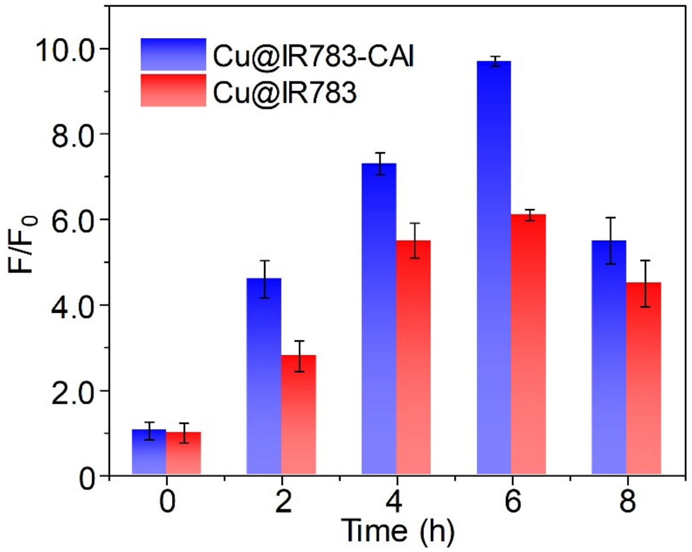

Figure S13. NIR-II fluorescence enhancement of CT26 cells incubated with Cu@IR783-CAI or Cu@IR783 for different times compared with initial.

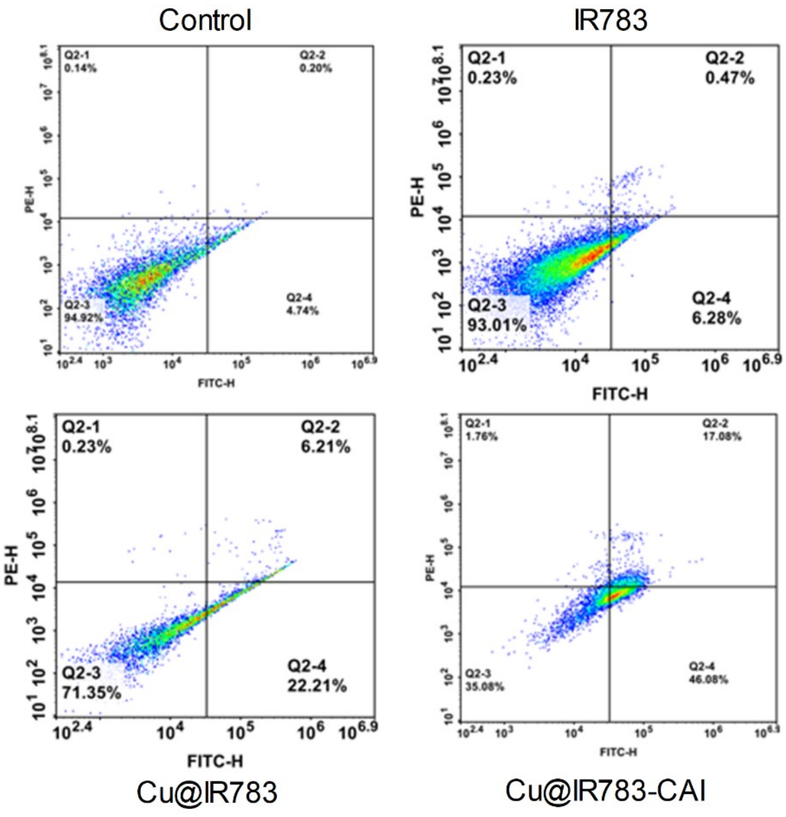

Figure S14. Flow cytometry analysis of viability of CT26 cells under different treatments.

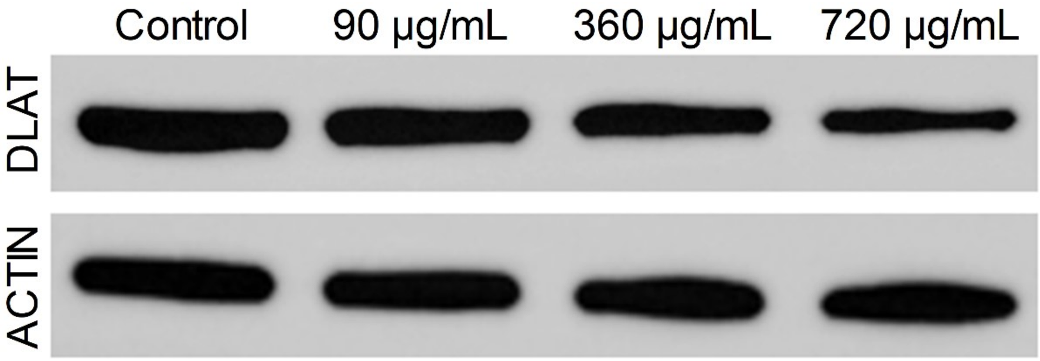

Figure S15. Western blotting of DLAT within CT26 cells treated with different concentrations of Cu@IR783-CAI.

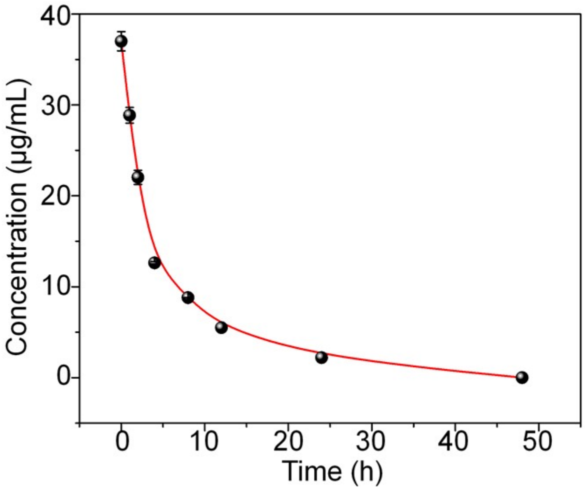

Figure S16. The blood concentration of Cu@IR783-CAI as a function of post-injection time.

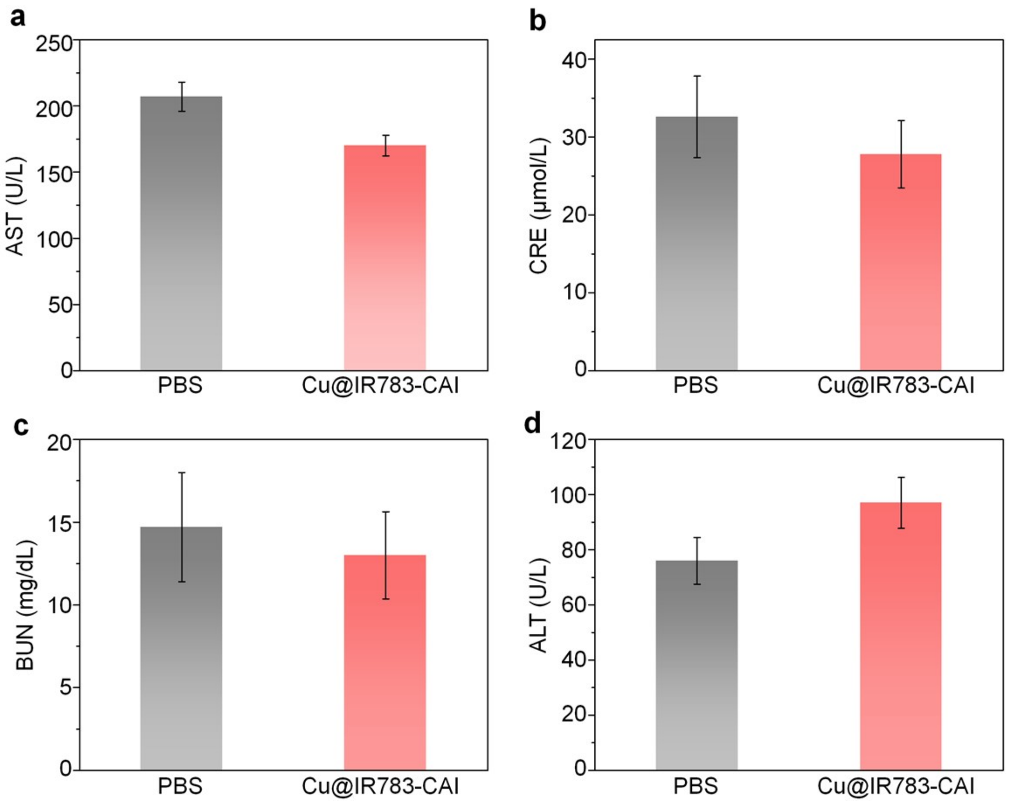

Figure S17. Biochemical analysis of blood collected from mice after different treatments. The error bars represent standard deviations of five different measurements (n = 5).
